# Correlation among *Streptococcus bovis*, endocarditis and septicemia in a patient with advanced colon cancer: a case report

**DOI:** 10.1186/1752-1947-7-185

**Published:** 2013-07-15

**Authors:** Chiara Abeni, Luigina Rota, Chiara Ogliosi, Paola Bertocchi, Pietro Berra Centurini, Alberto Zaniboni

**Affiliations:** 1 Department of Medical Oncology, Fondazione Poliambulanza, Via Bissolati 57, 25124, Brescia, Italy; 2 Department of Cardiology, Fondazione Poliambulanza, Via Bissolati 57, 25124, Brescia, Italy

**Keywords:** *Streptococcus bovis/gallolyiticus*, Endocarditis, Colon cancer

## Abstract

**Introduction:**

One of the bacterial agents that has been found to be associated with colorectal cancer is *Streptococcus bovis*, with 13% of infective endocarditis cases caused by this pathogenic species.

**Case presentation:**

We describe the case of a 57-year-old Caucasian man with infiltrating and ulcerating metastatic adenocarcinoma of the sigmoid colon. The patient was receiving second-line chemotherapy treatment and, on the eighth day of the second cycle, he developed a grade IV pancytopenia. We diagnosed a severe sepsis with positive blood cultures for *Streptococcus bovis/gallolyticus* with a secondary endocarditis.

**Conclusions:**

A recent study suggests that the majority of patients affected by colonic cancer have a *Streptococcus bovis/gallolyticus* colonization that becomes apparent as an overt infection only when immunosystem disorders or cardiac valve lesions occur. This correlation is important for involving more specialists in a correct and early diagnosis of this rare, but potentially fatal, complication.

## Introduction

The human gastrointestinal tract is colonized by different commensal bacterial species. This bacterial population prevents the invasion of pathogenic bacteria [1].

Among the commensal bacteria, *Streptococcus bovis* (*S. bovis*) is found in 2.5 to 15% of the population. Several studies have shown a correlation between the presence of *S. bovis* biotype I bacteremia and colon cancer.

In addition, 13% of infective endocarditis cases are caused by this pathogenic species [2,3].

The aim of this report is to present the clinical, diagnostic, and therapeutic aspects of a patient with advanced colon cancer who developed secondary neutropenia after antineoplastic treatment with subsequent onset of meningoencephalitis and endocarditis related to *S. bovis/gallolyticus* infection.

The clinical, diagnostic and therapeutic aspects will be discussed.

## Case presentation

A 57-year-old Caucasian man with no family history of neoplastic diseases and no comorbidities, after severe weight loss and abdominal pain, was examined by his general practitioner for tumor markers (carcinoembryonic antigen (CEA)=4448 and cancer antigen (CA)19.9=4728). His fecal occult blood test was positive and the biopsies obtained during a colonoscopy were positive for infiltrating and ulcerated poorly differentiated adenocarcinoma (G3) of the sigmoid colon.

An immunohistochemistry analysis revealed epidermal growth factor receptor (EGFR) positivity and molecular analysis showed that both KRAS and BRAF were not mutated.

A staging computed tomography (CT) scan showed liver, lung, and spleen metastases and the presence of peritoneal carcinomatosis. Because of the advanced stage of disease, no indication was given for surgical treatment. The patient underwent 10 cycles of chemotherapy according to the FOLFOX6 regimen (oxaliplatin 100mg/mq; leucovorin 400mg/mq; 5-fluorouracil 400mg/mq bolus followed by 5-fluorouracil 2400mg/mq by continuous infusion over 46 hours every two weeks) with markers reduction (CEA=681 and CA19.9=330), improved performance status and weight gain. His chemotherapy treatment was continued with capecitabine, 1000mg/mq twice a day, as a maintenance therapy for two additional months. After this treatment, a CT scan showed progression of the disease in the lungs and liver, and laboratory examinations showed an increase in the tumor markers (CEA=820 and CA19.9=507).

The patient started a second-line chemotherapy with cetuximab, 400mg/mq loading dose, and irinotecan 200mg/mq. On the eighth day of the second cycle the patient developed fever (39°C), diarrhea, and confusion and the neurological examination by the emergency service revealed severe opisthotonus and retroversion of the eyes. A brain CT scan was negative for ischemic events or secondary lesions and his electroencephalogram (EEG) was normal.

In the afternoon, the patient was admitted to the medical oncology unit, where he was diagnosed with severe sepsis with clinical involvement of the meningoencephalic system and grade IV pancyopenia (hemoglobin (Hb)=8.5g/dL, red blood cells (RBC)=2.8×10^6/mL, platelets (PLT)=31000mL, white blood cells (WBC)=300mL, neutrophils=100mL).

While waiting for blood culture results, he started empiric antibiotic therapy with 500mg levofloxacin administered intravenously every 12 hours and 200mg intravenous fluconazole once a day in association with granulocyte colony-stimulating factor (G-CSF). After five days, the pancytopenia resolved but the neurological disorder persisted.

**Figure 1:**
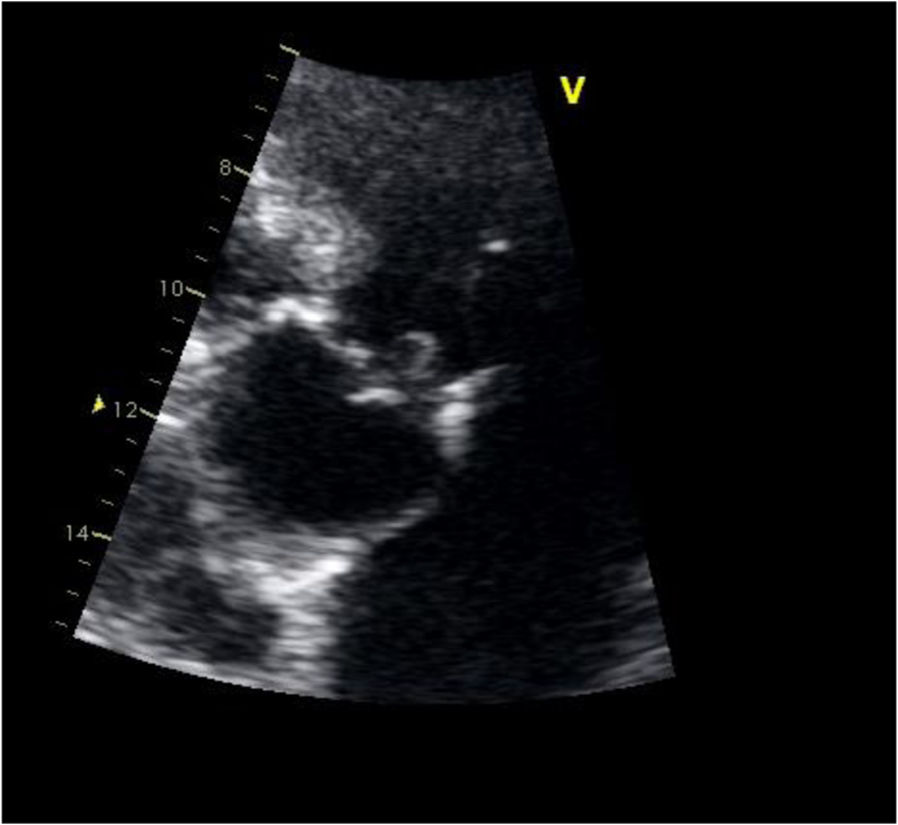
Endocardial vegetation stack on the right coronary cusp.

In the meantime, *Streptococcus gallolyticus subsp gallolyticus* was isolated in the blood cultures. Echocardiography showed a moderate aortic valve insufficiency due to an endocardial vegetation stack on the right coronary cusp (about 10mm in diameter) protruding in the outflow tract of the left ventricle (Figure 1). A cerebrospinal fluid sample was not taken due to the very low platelet count (5000/mL on the second day after admission).

Ampicillin and sulbactam (3g intravenous every six hours) were added to the antibiotic treatment.

On the fourteenth day, the patient’s neurological status improved and he exhibited only residual mild opisthotonus with no cognitive deficit.

A total body CT scan was performed on the third week after admission, which showed progressive disease, thus antineoplastic therapy was ceased.

## Discussion

The incidence of colon cancer with *S. bovis* endocarditis has been shown to be between 18 and 62% [4-10].

The co-occurrence of a bacterial endocarditis and colon carcinoma was first reported in 1951 [11] but the association of *S. bovis* and colorectal neoplasia was not recognized until 1974 [12]. *S bovis* was documented by Klein *et al*. as the pathogenic agent specifically related to the presence of a colon cancer in 1977 [13].

Bacteria are linked to cancer by two mechanisms: chronic inflammation and the production of carcinogenic metabolites [14].

*S. bovis/gallolyticus* has been reported to increase the production of inflammatory cytokines in the colonic mucosa of rats, suggesting a direct interaction between *S. bovis* and colonic mucosal cells, which is thought to lead to the development of colorectal cancer. It should be noted that in humans most colonic neoplasms associated with *S. bovis* bacteremia are ulcerated adenomas, a well-known precursor of invasive cancer [15-19].

In our clinical case, the neoplastic disease was already present when the patient developed bacteremia and endocarditis secondary to the infection with *S. bovis*. The infection occurred in the context of severe neutropenia. We assume that due to the critical condition of the patient, he developed endocarditis and possibly a meningoencephalitis as a result of the transition of the *S. bovis/gallolyticus* through the ulcerated tumoral lesion of the colon into the bloodstream.

A recent study suggested that the majority of patients affected by colonic cancer develop a silent infection, but it becomes apparent when immunosystem disorders or cardiac valve lesions occur [20].

Considering the correlation between *S. bovis/gallolyticus*, endocarditis and tumors, colorectal endoscopic evaluation would be useful in patients who have developed bacterial endocarditis caused by *S. bovis/gallolyticus* in order to identify silent colorectal cancer as early as possible.

## Conclusions

In these cases, the teamwork of several specialists (cardiologist, infectivologist, endoscopist, oncologist) remains the gold standard for an accurate diagnostic workup and prompt treatment decisions.

Therefore, understanding whether other cases like ours have occurred and what to do in such situations would be helpful.

In addition, the peculiar aspect of this case is the presence of a neurological involvement, which could be due to both the septic status and to a specific sensibility of the brain membrane to *Streptococcus bovis/gallolyticus*, albeit a cerebrospinal fluid (CSF) analysis was not done.

## Consent

Written informed consent was obtained from the patient for publication of this case report and any accompanying images. A copy of the written consent is available for review by the Editor-in-Chief of this journal.

## Competing interests

The authors declare that they have no competing interests.

## Authors’ contributions

AC, RL, BP, OC, BCP and ZA diagnosed and followed the patient. BCP performed and interpreted the echocardiography. AC, RL and OC prepared the manuscript and ZA evaluated the draft and suggested revisions. All authors read and approved the final manuscript.
